# Curative-Intent Chemoimmunotherapy in Stage IIB Triple-Negative Breast Cancer in a Patient on Chronic Peritoneal Dialysis: A Case Report and Literature Review

**DOI:** 10.7759/cureus.106064

**Published:** 2026-03-29

**Authors:** Maria Jamil, Samuel Srinivasan, Hiba Jabbour-Aida, Lisi Yuan, Javairia Jamil

**Affiliations:** 1 Hematology and Medical Oncology, Henry Ford Health System, Detroit, USA; 2 Internal Medicine, Henry Ford Health System, Detroit, USA; 3 Pathology, Henry Ford Health System, Detroit, USA; 4 Internal Medicine, University of Central Florida/HCA North Florida Hospital, Gainesville, USA

**Keywords:** carboplatin dosing, curative intent therapy, end-stage renal disease, keynote-522, neoadjuvant chemotherapy, peritoneal dialysis, stage iib breast cancer, triple-negative breast cancer

## Abstract

Triple-negative breast cancer (TNBC) is an aggressive subtype associated with higher recurrence rates and inferior survival compared with hormone receptor-positive disease. The addition of immune checkpoint inhibition to neoadjuvant chemotherapy has improved pathologic complete response (pCR) and event-free survival in early-stage TNBC and is now the standard of care for high-risk disease. However, patients with end-stage renal disease (ESRD) requiring dialysis were excluded from pivotal clinical trials, leaving limited evidence to guide treatment in this population. We report the case of a 41-year-old woman with clinical stage IIB (cT2N1M0) TNBC and ESRD on chronic peritoneal dialysis who received modified neoadjuvant chemoimmunotherapy based on the KEYNOTE-522 regimen. Chemotherapy dosing was individualized for renal failure, including flat-dose carboplatin and dose-reduced anthracycline and cyclophosphamide, while pembrolizumab was administered at standard dosing. She completed neoadjuvant therapy and 16 of 17 planned pembrolizumab cycles (final cycle omitted due to toxicity) with overall manageable adverse effects. Surgical pathology following lumpectomy and sentinel lymph node biopsy demonstrated pCR (residual cancer burden score of 0). At one-year follow-up, she remains without evidence of recurrence. This case demonstrates the feasibility of delivering curative-intent TNBC chemoimmunotherapy in a patient undergoing peritoneal dialysis and highlights the importance of multidisciplinary coordination in managing malignancy in patients with advanced renal disease.

## Introduction

Triple-negative breast cancer (TNBC) accounts for approximately 10-20% of invasive breast cancers and is defined by the absence of estrogen receptor, progesterone receptor, and human epidermal growth factor receptor 2 expression [1]. Compared with hormone receptor-positive subtypes, TNBC is associated with higher histologic grade, increased rates of visceral metastasis, and inferior survival outcomes [1,2]. Recurrence risk is greatest within the first three to five years after diagnosis, underscoring the importance of effective early systemic therapy [2].

Neoadjuvant chemotherapy has long been central to the management of stage II-III TNBC due to its relative chemosensitivity and the prognostic significance of pathologic complete response (pCR) [3]. Achieving pCR is strongly associated with improved long-term event-free and overall survival [3]. The phase III KEYNOTE-522 trial demonstrated that adding pembrolizumab to carboplatin and paclitaxel, followed by anthracycline and cyclophosphamide, significantly increased pCR rates and improved event-free survival in early-stage TNBC, establishing this regimen as the standard of care for high-risk disease [4].

Patients with severe renal impairment or end-stage renal disease (ESRD) requiring dialysis were excluded from KEYNOTE-522 and most prospective breast cancer trials [4]. Consequently, there is limited evidence to guide the administration of chemoimmunotherapy in dialysis-dependent patients. ESRD affects hundreds of thousands of individuals in the United States, with dialysis serving as the primary renal replacement modality [5]. While most patients undergo hemodialysis, a smaller proportion utilize peritoneal dialysis (PD), which differs in drug clearance characteristics and pharmacokinetics [5,6].

The administration of systemic therapy in patients receiving PD presents unique challenges due to altered drug metabolism, variable dialysis clearance, and comorbidity burden. Data regarding carboplatin, anthracyclines, cyclophosphamide, and immune checkpoint inhibitors in PD patients, particularly in the curative-intent setting, remain sparse. We report the case of a patient with stage IIB TNBC and ESRD on chronic PD who received modified KEYNOTE-522-based therapy and achieved pCR. There are no established dosing strategies or prospective data guiding curative-intent chemoimmunotherapy in patients undergoing PD.

## Case presentation

A 41-year-old woman with a history of ESRD secondary to long-standing type 1 diabetes mellitus and hypertension presented after an abnormal screening mammogram revealed a suspicious left breast mass. She had been maintained on automated PD using a cycler for approximately eight hours nightly (2 L exchanges, five cycles, six days per week), with negligible residual renal function. Her comorbidities included diabetic peripheral neuropathy and hypertension. Her Eastern Cooperative Oncology Group (ECOG) performance status at diagnosis was 1.

**Figure 1:**
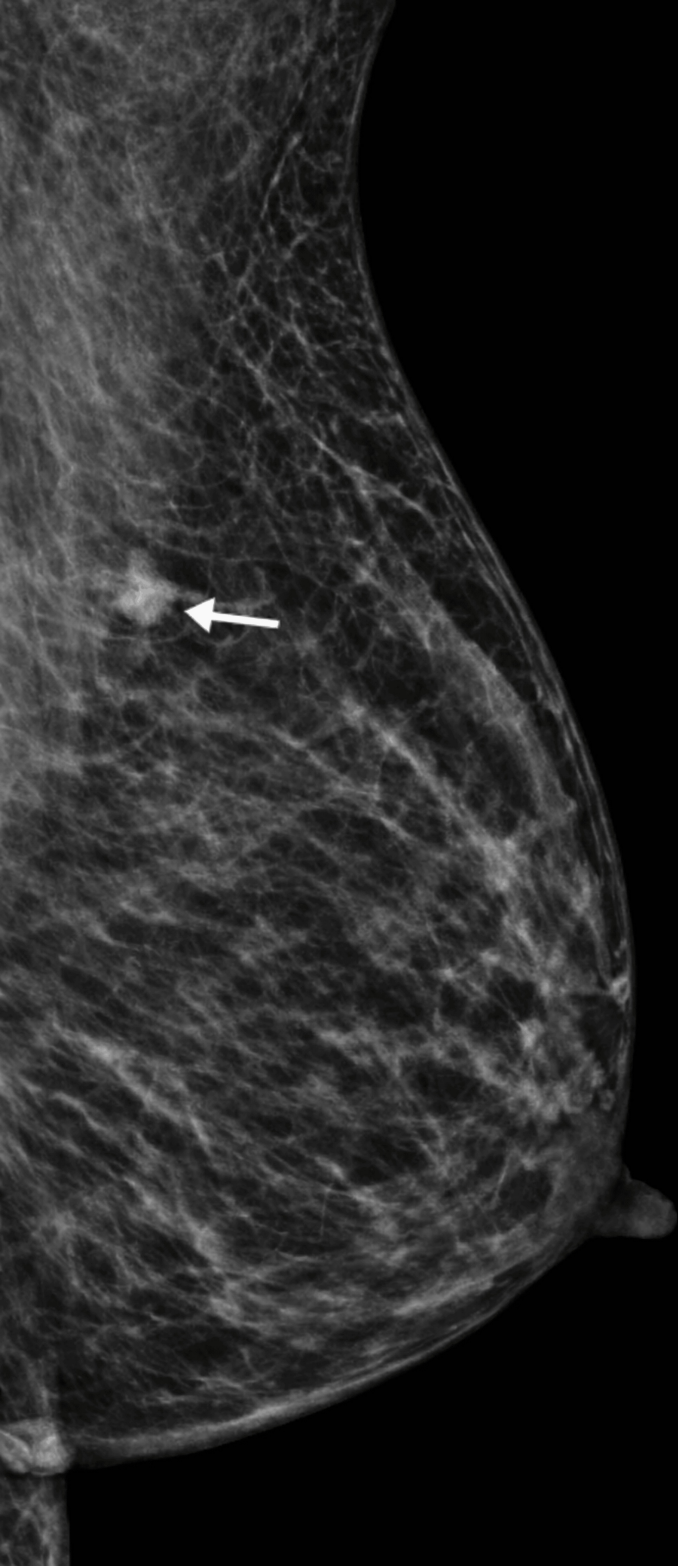
Left mediolateral oblique mammogram demonstrating an irregular spiculated mass in the left breast (arrow).

**Figure 2:**
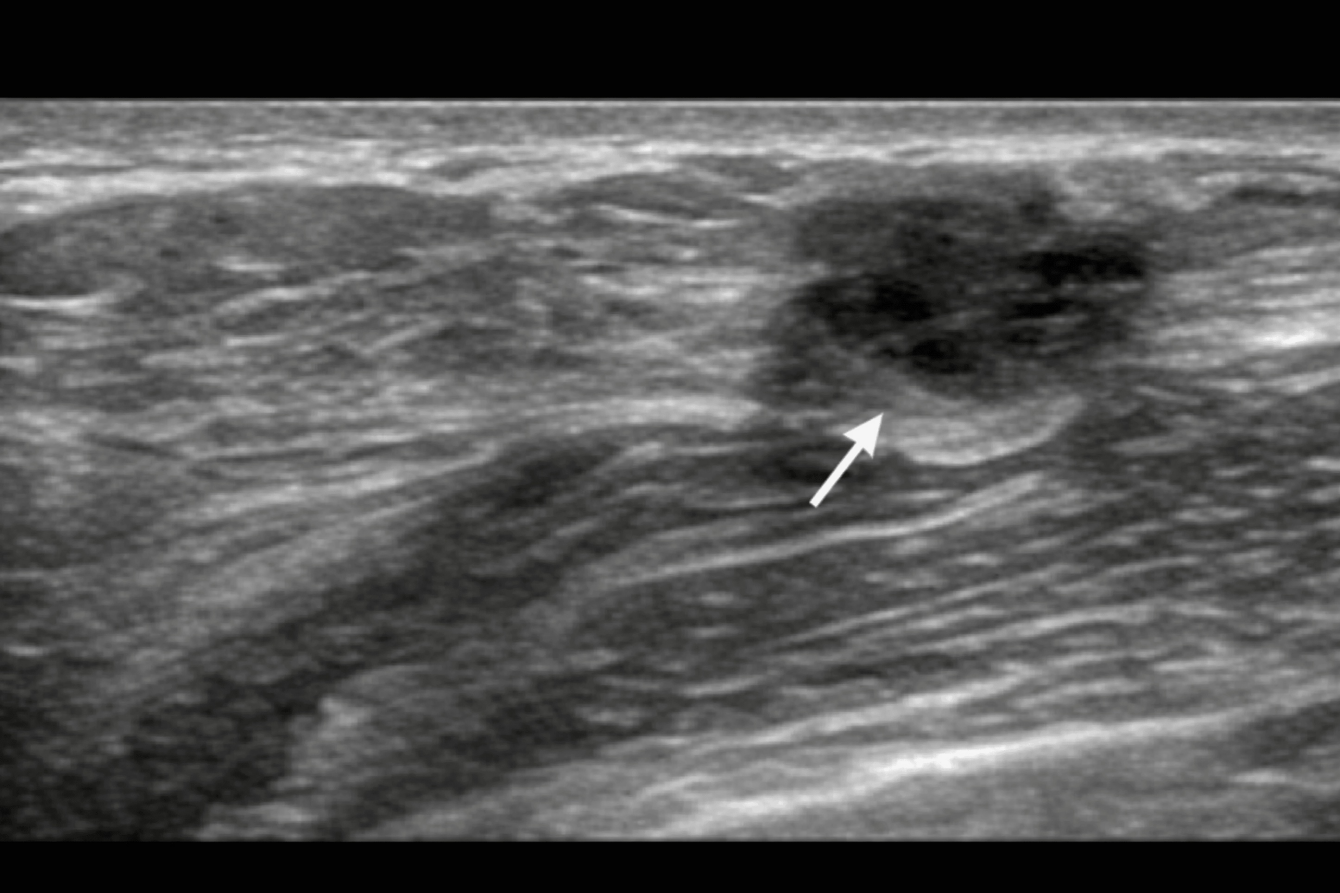
Targeted breast ultrasound confirming a corresponding irregular hypoechoic mass at the 2 o’clock position.

**Figure 3:**
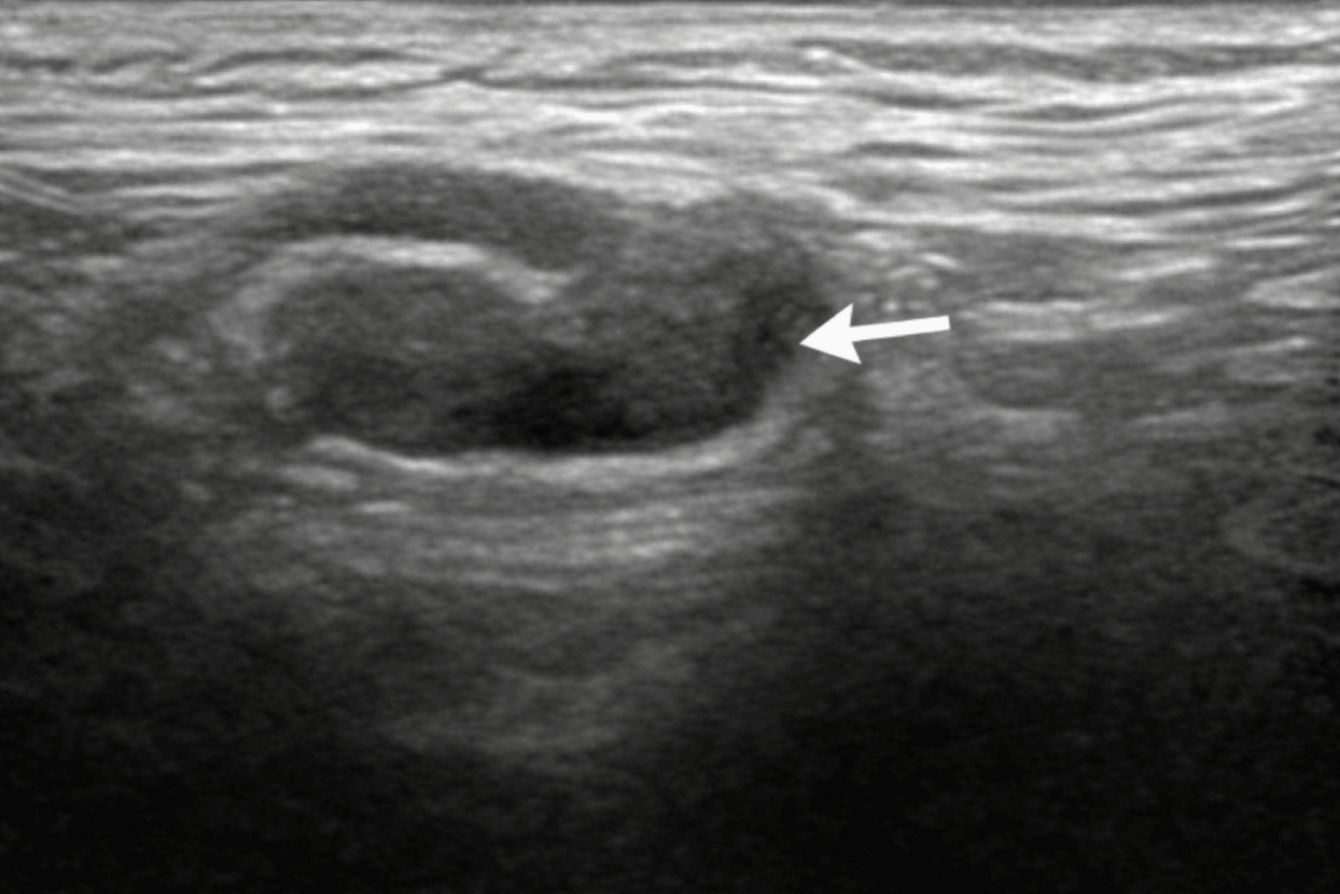
Targeted ultrasound of the left axilla demonstrating an abnormal lymph node with cortical thickening and loss of fatty hilum (arrow).

Diagnostic mammography demonstrated a 2.5 cm irregular spiculated mass in the left breast (Figure 1). Targeted breast ultrasound confirmed a corresponding irregular hypoechoic mass (Figure 2) with associated abnormal axillary lymphadenopathy (Figure 3).

**Figure 4:**
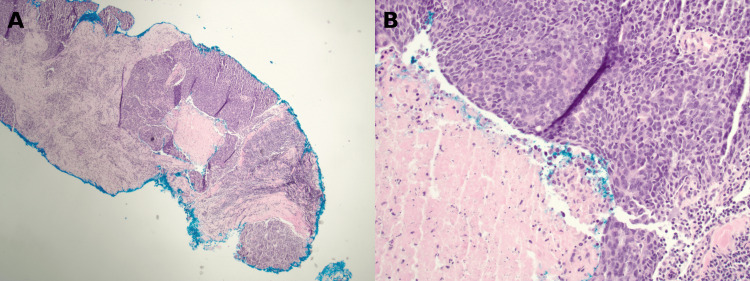
Histopathology of the breast core biopsy. (A) Low-power hematoxylin and eosin (H&E) stain demonstrating invasive carcinoma infiltrating the breast stroma with surrounding inflammatory cells. (B) High-power H&E view showing pleomorphic malignant cells with tumor necrosis, consistent with high-grade invasive ductal carcinoma.

**Figure 5:**
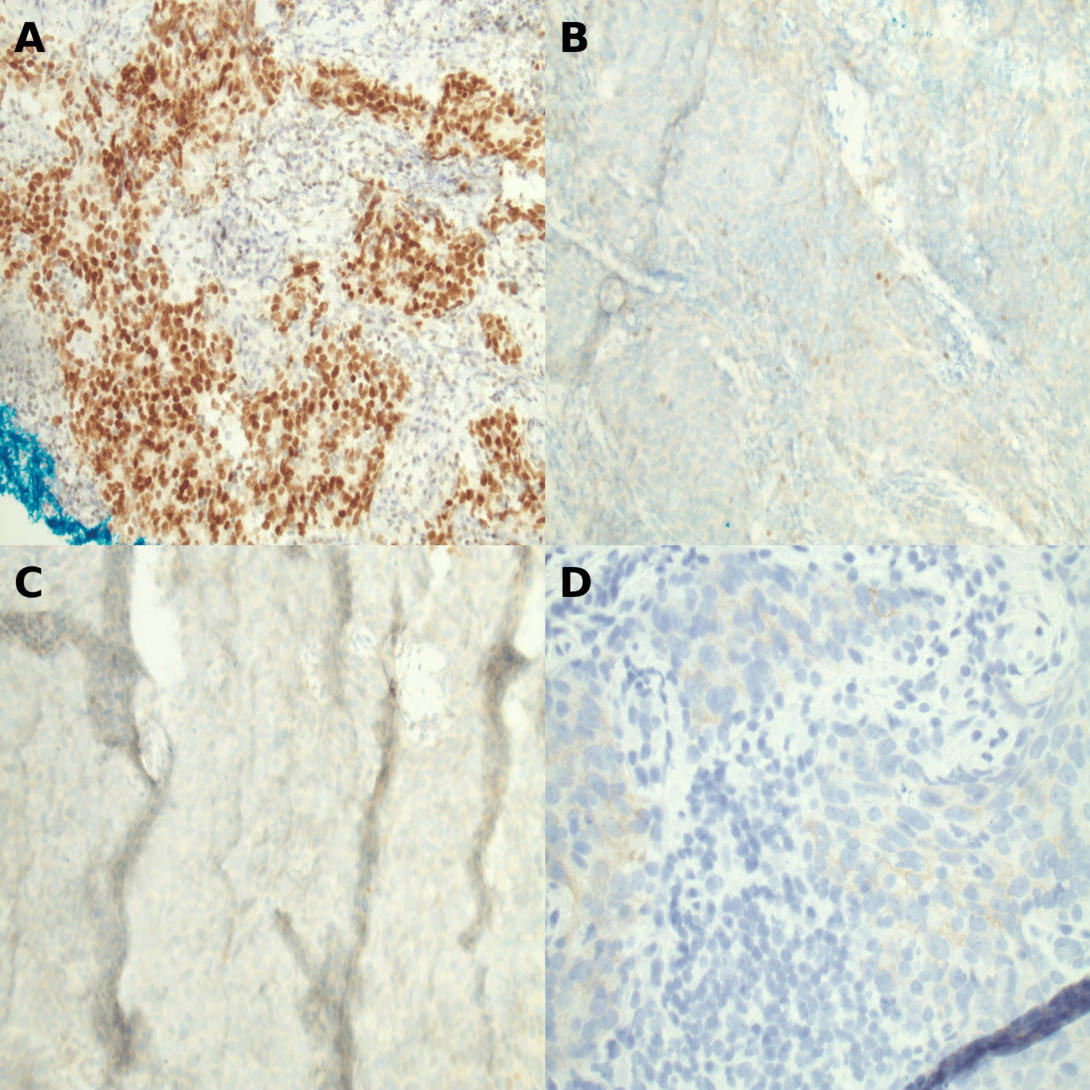
Immunohistochemical characterization of the breast tumor. (A) GATA-3 immunostain demonstrating strong nuclear positivity in tumor cells, supporting mammary epithelial origin. (B) Estrogen receptor immunostain showing negative staining (<1%). (C) Progesterone receptor immunostain showing negative staining. (D) Human epidermal growth factor receptor 2 immunostain showing negative staining (1+). These findings confirm a triple-negative breast cancer phenotype.

Ultrasound-guided core needle biopsy of the breast lesion demonstrated high-grade invasive ductal carcinoma (Figures 4A, 4B). Histologic examination showed infiltrative malignant epithelial cells with marked nuclear pleomorphism and areas of tumor necrosis. Immunohistochemical staining demonstrated strong nuclear positivity for GATA-3, supporting mammary origin (Figure 5A). Receptor testing confirmed triple-negative disease (estrogen receptor <1%, progesterone receptor negative, human epidermal growth factor receptor 2 1+), with a Ki-67 proliferation index >70% (Figures 5B-5D).

**Figure 6:**
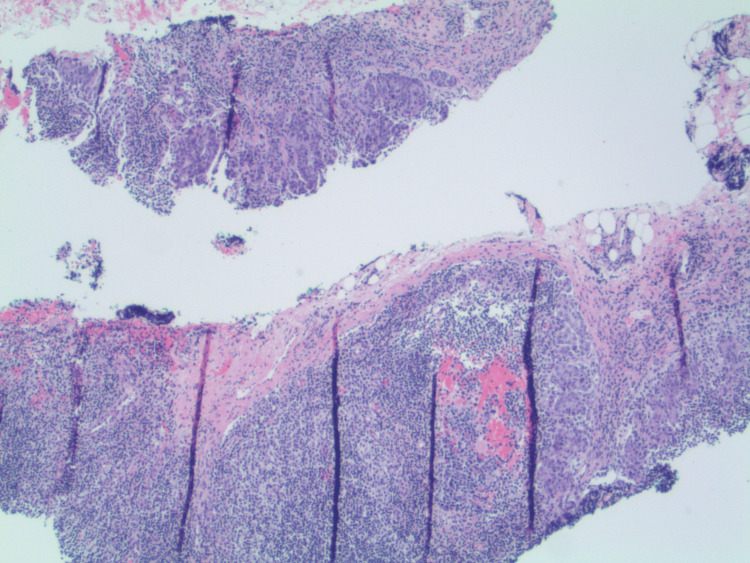
Left axillary lymph node biopsy demonstrating metastatic carcinoma (hematoxylin and eosin, 40× magnification). The lymph node architecture is effaced by infiltrating nests and sheets of atypical epithelial cells consistent with metastatic carcinoma.

Core needle biopsy of the left axillary lymph node demonstrated metastatic carcinoma, confirming nodal involvement (Figure 6).

**Figure 7:**
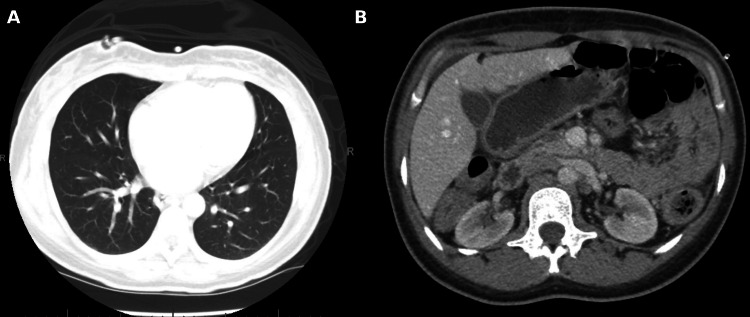
Contrast-enhanced axial CT of the chest (A) and abdomen (B) demonstrating no evidence of pulmonary or hepatic metastatic disease at diagnosis, consistent with clinical stage cT2N1M0.

Contrast-enhanced CT of the chest and abdomen demonstrated no evidence of distant metastatic disease (Figure 7). The tumor was clinically staged as cT2N1M0, corresponding to stage IIB disease.

Given her high-risk, early-stage TNBC with nodal involvement, neoadjuvant chemoimmunotherapy was recommended. Due to the absence of trial data in dialysis-dependent patients, a multidisciplinary discussion involving oncology, nephrology, and pharmacy was undertaken to individualize treatment.

Baseline laboratory values were notable for anemia (hemoglobin ~10-11 g/dL), markedly elevated creatinine consistent with ESRD, and hypoalbuminemia (~3.0 g/dL), reflecting her underlying chronic illness. Her body surface area was 1.78 m².

She was initiated on neoadjuvant therapy consisting of carboplatin, paclitaxel, and pembrolizumab. Carboplatin was administered as a fixed dose of 50 mg weekly, representing a substantial reduction compared with conventional area under the curve (AUC)-based dosing, given the absence of reliable renal clearance and unpredictable pharmacokinetics in PD [7,8]. Paclitaxel was administered at approximately 80 mg/m² weekly (144 mg), consistent with standard dosing [9]. Pembrolizumab was administered at the full standard dosing without adjustment [10]. Chemotherapy administration was not specifically timed relative to dialysis exchanges, and her PD regimen remained unchanged throughout treatment.

After completion of four cycles of carboplatin and paclitaxel with pembrolizumab, she transitioned to doxorubicin and cyclophosphamide with pembrolizumab. Both agents were administered at 50% of standard dosing due to concerns regarding metabolite accumulation and toxicity risk in ESRD [11,12].

She tolerated the therapy well overall. Toxicities included grade 1-2 fatigue and mild cytopenias without the need for transfusion or hospitalization. Her baseline diabetic neuropathy remained stable, and no immune-related adverse events were observed.

**Figure 8:**
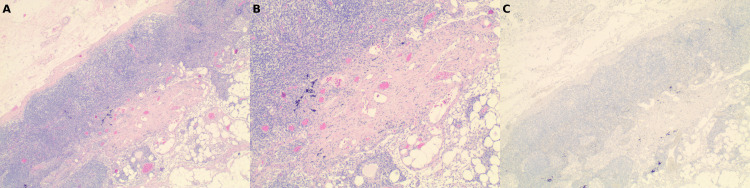
Sentinel lymph node after neoadjuvant chemoimmunotherapy demonstrating pathologic complete response. (A) Sentinel lymph node demonstrating biopsy site changes and therapy-related fibrosis without residual metastatic carcinoma (hematoxylin and eosin (H&E), 40× magnification). (B) Higher magnification showing stromal fibrosis, histiocytic infiltration, and treatment effect without viable tumor cells (H&E, 100× magnification). (C) Immunohistochemical stain for pancytokeratin (AE1/AE3) negative for residual epithelial tumor cells, confirming absence of metastatic carcinoma (immunohistochemical stain, 40× magnification).

Post-neoadjuvant imaging demonstrated a marked reduction in the primary tumor and resolution of axillary lymphadenopathy. She underwent lumpectomy with sentinel lymph node biopsy approximately six months after treatment initiation. Final pathology demonstrated pCR (residual cancer burden (RCB) score of 0) with no residual invasive carcinoma and no residual nodal disease [13]. Histopathologic evaluation confirmed treatment effect without viable tumor cells (Figures 8A-8C), with negative pancytokeratin staining.

At the one-year follow-up, the patient remains without evidence of recurrence. She has returned to her baseline functional status and continues routine outpatient activities. She remains on PD without change in modality or schedule. Dialysis adequacy has been preserved (Kt/V 2.03), with no evidence of peritoneal membrane dysfunction or dialysis-related complications attributable to systemic therapy.

## Discussion

The management of early-stage TNBC in patients with ESRD requiring dialysis presents significant therapeutic challenges due to the absence of prospective clinical data and complex pharmacokinetic considerations. Dialysis-dependent patients were excluded from KEYNOTE-522 [4], requiring clinicians to extrapolate from limited pharmacologic data and observational experience. Published data on chemoimmunotherapy in dialysis-dependent TNBC patients are extremely limited. Prior reports have primarily described chemotherapy alone in hemodialysis patients, with minimal data in PD and none incorporating immune checkpoint inhibitors in the curative-intent setting.

Carboplatin dosing is particularly challenging in ESRD. It is primarily renally excreted and traditionally dosed using the Calvert formula, which incorporates glomerular filtration rate to target a specific area under the concentration-time curve [7]. In ESRD, negligible filtration renders AUC-based dosing unreliable. Available data in renal insufficiency populations demonstrate increased toxicity risk and emphasize individualized dose adjustment [8]. Pharmacokinetic data in PD are extremely limited. PD provides continuous but lower-efficiency solute clearance compared with intermittent hemodialysis, and drug removal depends on molecular weight and protein binding characteristics [6]. Given these uncertainties, a conservative flat-dose approach was selected to mitigate severe myelosuppression risk.

Paclitaxel undergoes hepatic metabolism via CYP2C8 and CYP3A4 with minimal renal excretion [9]. Renal impairment does not significantly alter its clearance, and high protein binding limits dialysis removal [9], supporting standard dosing in this case.

Doxorubicin is hepatically metabolized, though metabolite accumulation may occur in renal dysfunction, potentially increasing hematologic and cardiac toxicity [11]. Cyclophosphamide is hepatically activated, with partially renally excreted metabolites; severe renal impairment may increase systemic exposure and myelosuppression risk [12]. In the absence of standardized dosing guidance for dialysis-dependent patients receiving curative-intent therapy, dose reductions were implemented to balance efficacy and safety.

Pembrolizumab is a humanized IgG4 monoclonal antibody not renally cleared and not expected to be removed by dialysis [10]. Limited observational data suggest immune checkpoint inhibitors may be administered in patients with renal dysfunction without substantially increased immune-related toxicity [14], though prospective data remain lacking. In this patient, pembrolizumab was well tolerated.

pCR is a strong prognostic marker in TNBC. The CTNeoBC pooled analysis demonstrated significantly improved event-free and overall survival among patients achieving pCR [3]. The achievement of RCB-0 in this case suggests that dose-modified therapy maintained meaningful antitumor efficacy despite dialysis dependence.

This report is limited by its single-patient design and absence of formal pharmacokinetic measurements. Long-term outcomes beyond one year remain unknown. Nevertheless, it contributes to the limited literature regarding curative-intent chemoimmunotherapy in patients undergoing PD.

## Conclusions

This case demonstrates that modified neoadjuvant chemoimmunotherapy based on the KEYNOTE-522 regimen can be administered safely and effectively in a patient with stage IIB TNBC undergoing chronic PD. With individualized dose adjustments and multidisciplinary monitoring, the patient achieved pCR without significant toxicity. This case supports the cautious use of standard-of-care regimens in dialysis-dependent patients when cure is the intent. Although prospective data are lacking, dialysis dependence alone should not automatically preclude standard-of-care therapy. Larger studies are needed to better define optimal dosing strategies and long-term outcomes in this population.

## References

[1] Foulkes WD, Smith IE, Reis-Filho JS (2010). Triple-negative breast cancer. N Engl J Med.

[2] Dent R, Trudeau M, Pritchard KI (2007). Triple-negative breast cancer: clinical features and patterns of recurrence. Clin Cancer Res.

[3] Cortazar P, Zhang L, Untch M (2014). Pathological complete response and long-term clinical benefit in breast cancer: the CTNeoBC pooled analysis. Lancet.

[4] Schmid P, Cortes J, Pusztai L (2020). Pembrolizumab for early triple-negative breast cancer. N Engl J Med.

[5] (2026). USRDS 2023 Annual Data Report: epidemiology of kidney disease in the United States. National Institute of Diabetes and Digestive and Kidney Diseases.

[6] Mujais S, Story K (2006). Peritoneal dialysis in the US: evaluation of outcomes in contemporary cohorts. Kidney Int Suppl.

[7] Calvert AH, Newell DR, Gumbrell LA (1989). Carboplatin dosage: prospective evaluation of a simple formula based on renal function. J Clin Oncol.

[8] Janus N, Launay-Vacher V, Byloos E (2010). Cancer and renal insufficiency results of the BIRMA study. Br J Cancer.

[9] Scripture CD, Figg WD, Sparreboom A (2005). Paclitaxel chemotherapy: from empiricism to a mechanism-based formulation strategy. Ther Clin Risk Manag.

[10] (2026). KEYTRUDA (pembrolizumab) prescribing information. Merck & Co., Inc.

[11] Minotti G, Menna P, Salvatorelli E, Cairo G, Gianni L (2004). Anthracyclines: molecular advances and pharmacologic developments in antitumor activity and cardiotoxicity. Pharmacol Rev.

[12] Juma FD, Rogers HJ, Trounce JR (1981). Effect of renal insufficiency on the pharmacokinetics of cyclophosphamide and some of its metabolites. Eur J Clin Pharmacol.

[13] Symmans WF, Peintinger F, Hatzis C (2007). Measurement of residual breast cancer burden to predict survival after neoadjuvant chemotherapy. J Clin Oncol.

[14] Cortazar FB, Marrone KA, Troxell ML (2016). Clinicopathological features of acute kidney injury associated with immune checkpoint inhibitors. Kidney Int.

